# Rare Instances of Culture-Positive Dengue-Related Panophthalmitis: A Case Series

**DOI:** 10.7759/cureus.63948

**Published:** 2024-07-06

**Authors:** Chaitanya Modgil, Renu Magdum, Iqra Mushtaq

**Affiliations:** 1 Ophthalmology, Dr. D. Y. Patil Medical College, Hospital & Research Centre, Pune, IND

**Keywords:** panophthalmitis, evisceration surgery, inflammation, haemorrhage, dengue fever

## Abstract

Panophthalmitis is an exceptionally rare but severe ocular complication of dengue fever, which is currently a significant health concern in parts of India. It is a purulent inflammation encompassing all structures of the globe (choroid, retina, vitreous fluid, aqueous fluid, cornea, sclera, and conjunctiva) along with surrounding orbital and periorbital structures. This case series highlights the occurrence of panophthalmitis in three patients diagnosed with dengue, who were aged 35, 50, and 75 years. Despite aggressive medical management, including intravenous antibiotics, the patients were ultimately scheduled for evisceration surgery due to the extreme severity of the condition. Healthcare providers must be aware of the potential ocular complications in dengue cases and diagnose them promptly. While ocular involvement in dengue is rare, this case series emphasizes the importance of recognizing ocular manifestations in dengue patients, as early diagnosis and prompt intervention can prevent severe complications.

## Introduction

Dengue fever is an acute febrile illness caused by a Flavivirus transmitted through the bite of the female Aedes aegypti mosquito [1,2]. Clinically, dengue fever is characterized by fever, arthralgia, headache, and a maculopapular rash [3]. It can be potentially life-threatening due to severe hemorrhagic complications, substantial capillary leakage leading to dengue shock, and potential involvement of vital organs [1]. While the exact mechanisms underlying the development of ophthalmic complications in dengue fever are not thoroughly elucidated, several studies have suggested that an immune-mediated process may play a significant role in this phenomenon [4].

Ocular inflammation and hemorrhagic complications are rare but known manifestations of dengue [1]. The spectrum of ocular conditions ranges from anterior to posterior uveitis and may include sub-conjunctival hemorrhage during the acute phase of the illness [2]. Additionally, dengue-associated ocular complications can lead to sight-threatening issues such as serous choroidal effusions, as well as dengue-associated maculopathy, including retinal hemorrhages and macular edema [2,5,6]. Panophthalmitis, a severe ocular condition, is rarely observed in the context of dengue hemorrhagic fever [1]. Intravenous administration of antibiotics and systemic corticosteroids should be considered in all instances of panophthalmitis [7]. We present a case series to explore the rare instances of culture-positive unilateral panophthalmitis in the context of dengue fever, a vision-threatening association.

## Case presentation

Case 1

**Figure 1:**
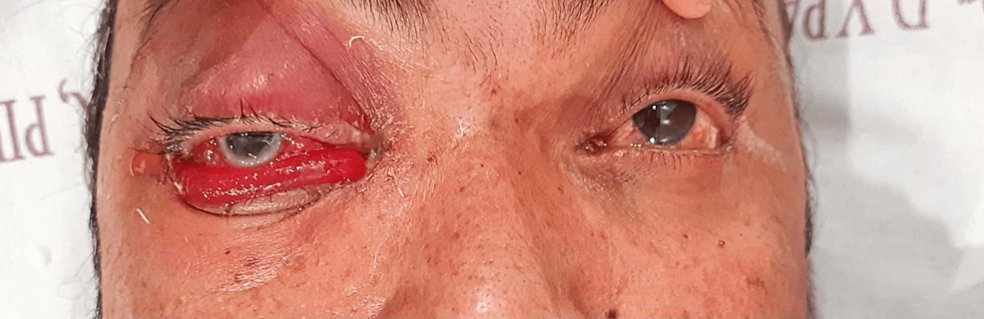
Image showing the presence of severe chemosis with lid edema with corneal haze and ring stromal infiltrates in the cornea; a flat anterior chamber was also present with a non-reactive pupil

A 35-year-old male presented with a one-week history of high-grade fever and three days of vomiting. He had tested positive for dengue NS1 antigen and had a low platelet count of 47,000/mm^3^ for which he received symptomatic treatment, including a random donor platelet transfusion. On the third day of his hospital stay, the patient developed severe retro-orbital pain and periorbital swelling with associated discharge over his right eye, which did not respond to medications (Figure 1).

**Figure 2:**
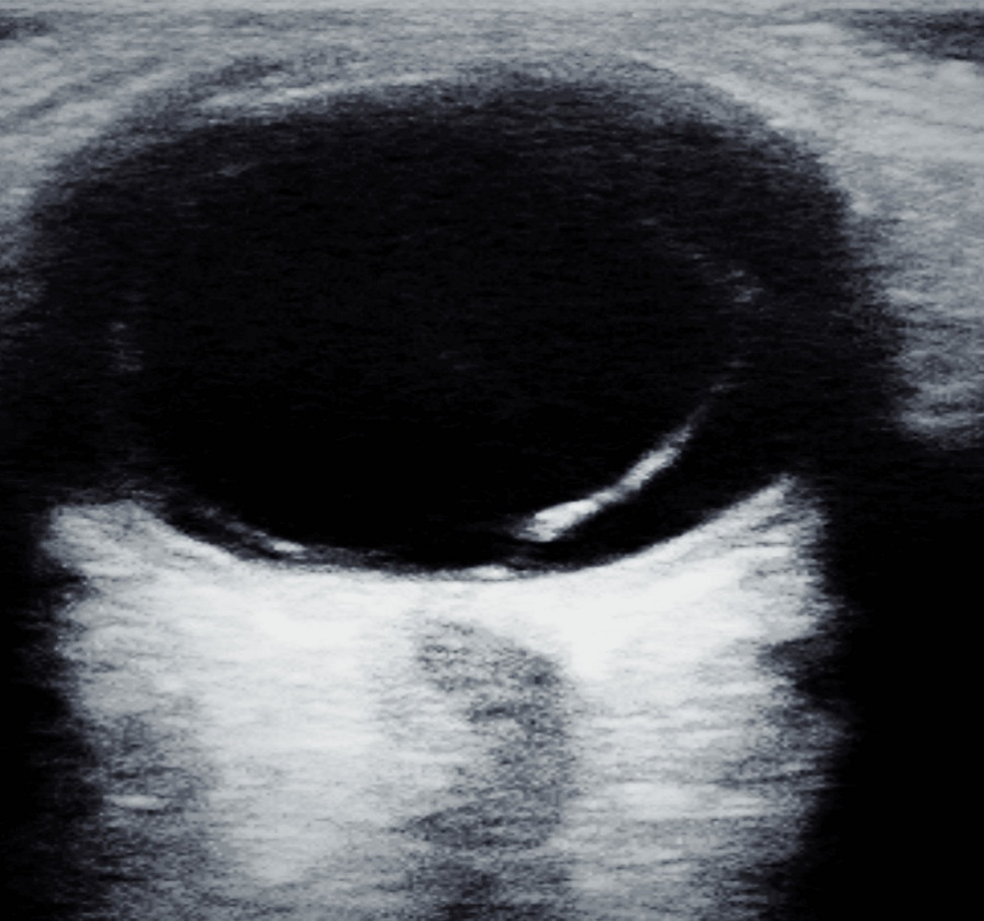
Ultrasound B-scan findings The image showed scleral thickening and the presence of membrane in a dome-shaped manner suggestive of sub-retinal fluid

**Figure 3:**
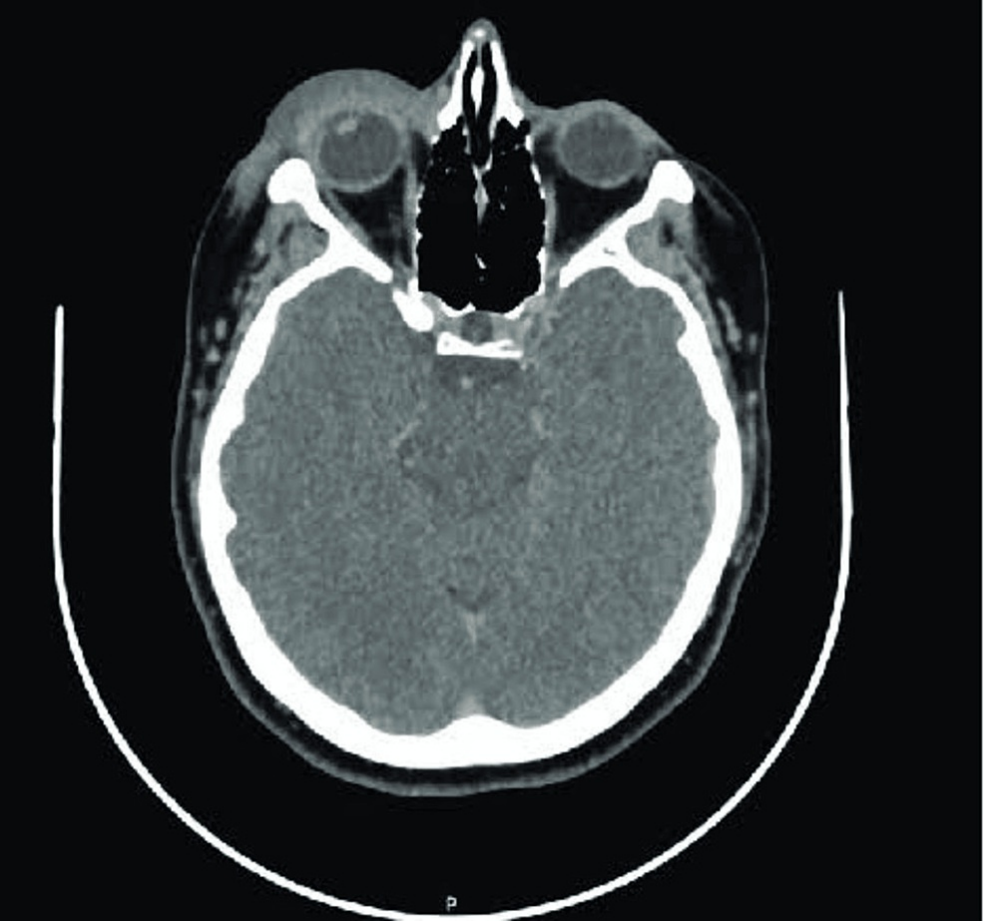
CT scan of the brain The image showed proptosis with diffuse soft tissue thickening anterior to the right orbital septum with thickening of the walls of the right eye suggestive of the thickening of ocular coats CT: computed tomography

**Figure 4:**
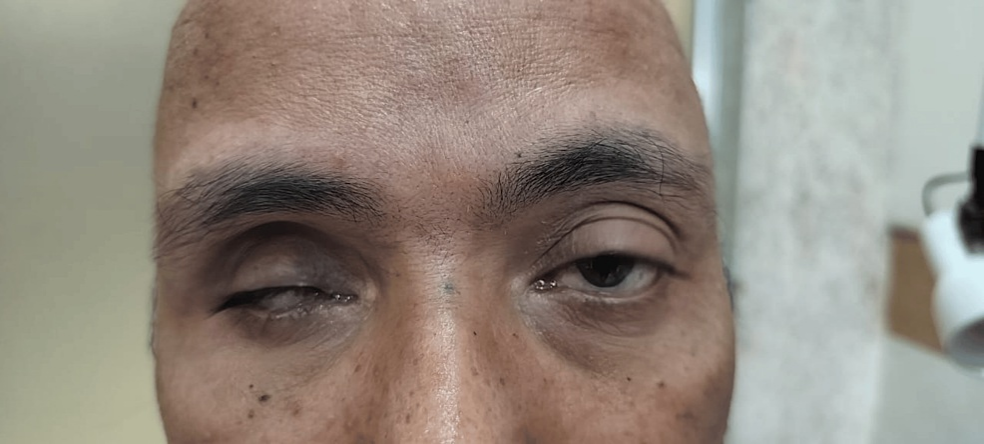
Image showing the eviscerated eye with conformer in situ

Examination revealed vision with no perception of light, severe chemosis, and congestion in the affected eye. Additional findings included a non-reactive pupil, corneal haziness, conjunctival congestion, stromal ring infiltrates, a flat anterior chamber, restricted extraocular movement, and digitally hard intraocular pressure. Fundoscopy was not possible due to the hazy cornea. A culture swab from the affected eye revealed bacillus cereus, while a CT scan of the brain and orbit showed right eye proptosis and thickening of ocular coats (Figure 2). An ultrasound B-scan showed scleral thickening, and multiple dome-shaped membranes indicative of sub-retinal fluid beneath a detached retina (Figure 3). The patient was started on broad-spectrum intravenous antibiotics and he underwent evisceration surgery for the affected eye, which was uneventful. A conformer prosthesis was inserted in place of the eviscerated eye, and the patient was discharged in stable condition after completing a one-week course of intravenous antibiotics (Figure 4).

Case 2

**Figure 5:**
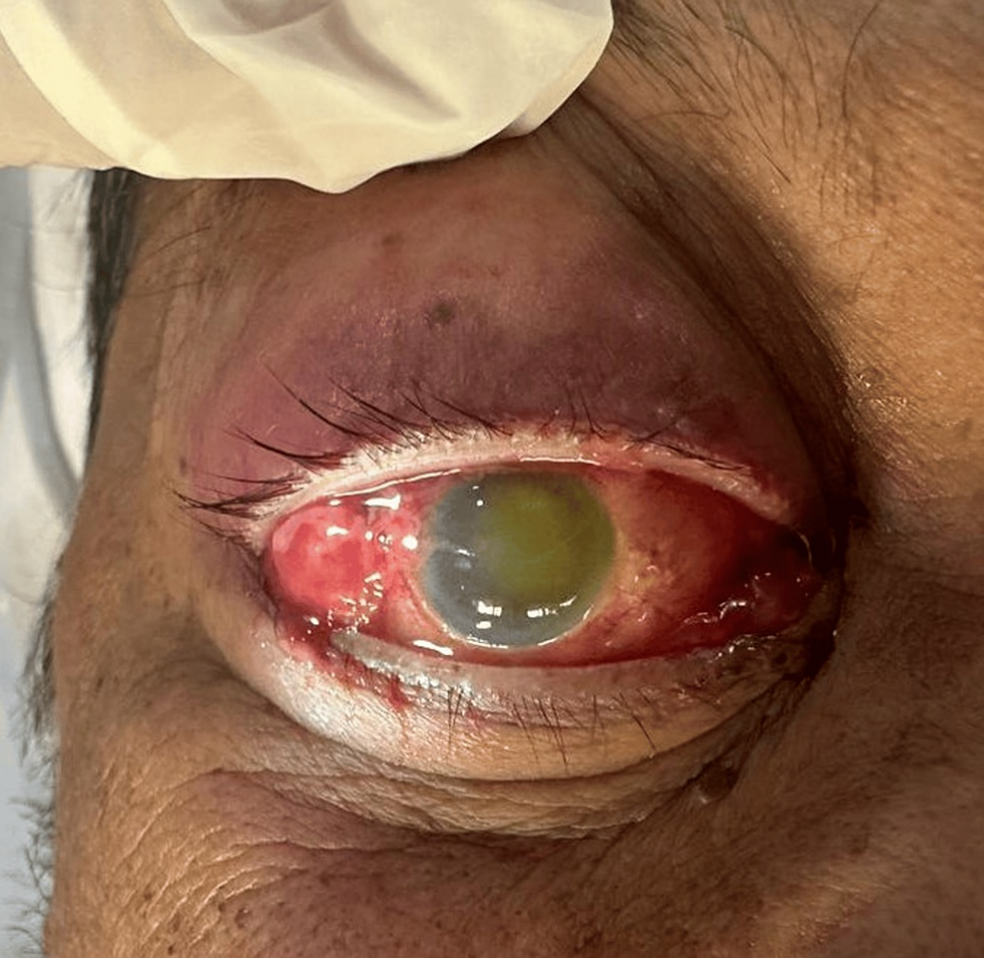
Image showing the presence of severe chemosis with corneal haze and ring stromal infiltrates in the cornea; a flat anterior chamber was also present with a non-reactive pupil

A 50-year-old seropositive male diagnosed with dengue three days back developed pain and swelling in his right eye. Upon admission, initial hematological analysis revealed a platelet count of 44,000/mm³, and the presence of NS1 antigen confirmed a serological diagnosis of dengue fever. The patient was commenced on supportive therapy, which included the administration of intravenous colloids, broad-spectrum intravenous antibiotics, and platelet transfusions. Two days after hospitalization, the patient developed ocular symptoms of severe pain and sudden progressive swelling in his right eye, which did not respond to medications. Examination revealed vision with no perception of light and a non-reactive pupil, along with severe chemosis, complete restriction of extraocular movements, and severe proptosis. There was a diffuse stromal haze, ring infiltrates in the cornea, and a flat anterior chamber. Indirect ophthalmoscopy could not be performed due to the hazy cornea (Figure 5).

**Figure 6:**
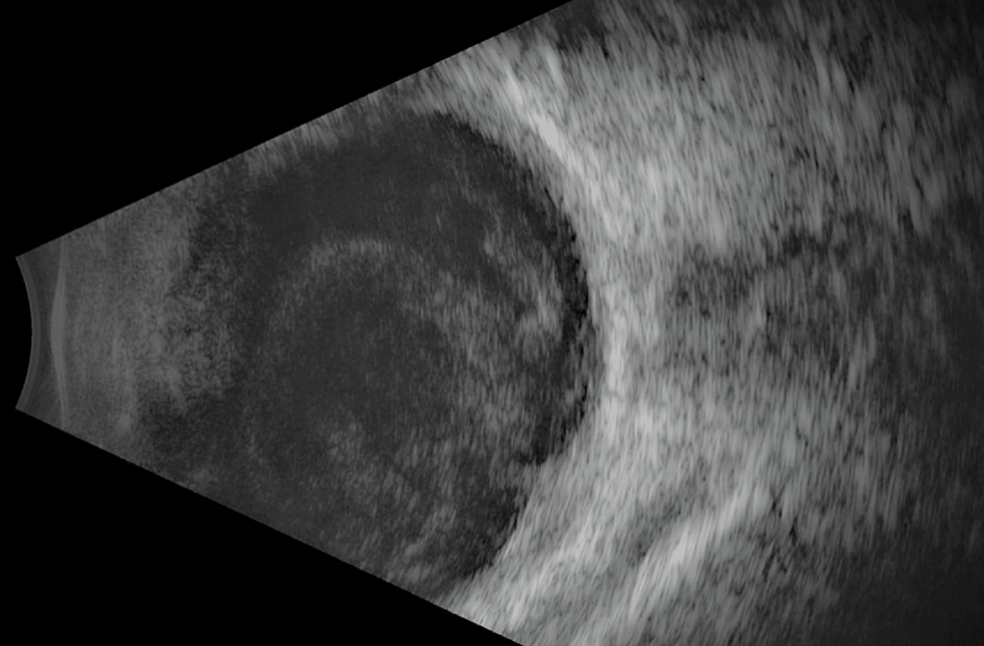
Ultrasound B-scan findings The image showed scleral thickening with the presence of vitreous opacities

The other eye was normal. A culture swab of the affected eye revealed Staphylococcus aureus. A B-scan of the affected eye showed vitreous opacities and scleral thickening suggestive of panophthalmitis (Figure 6). The patient received symptomatic treatment for dengue, including intravenous fluids and broad-spectrum antibiotics. He was eventually scheduled for evisceration surgery. Evisceration was done with a prosthesis in situ and the patient was discharged in stable condition on broad-spectrum antibiotics and advised to receive weekly follow-ups.

Case 3

**Figure 7:**
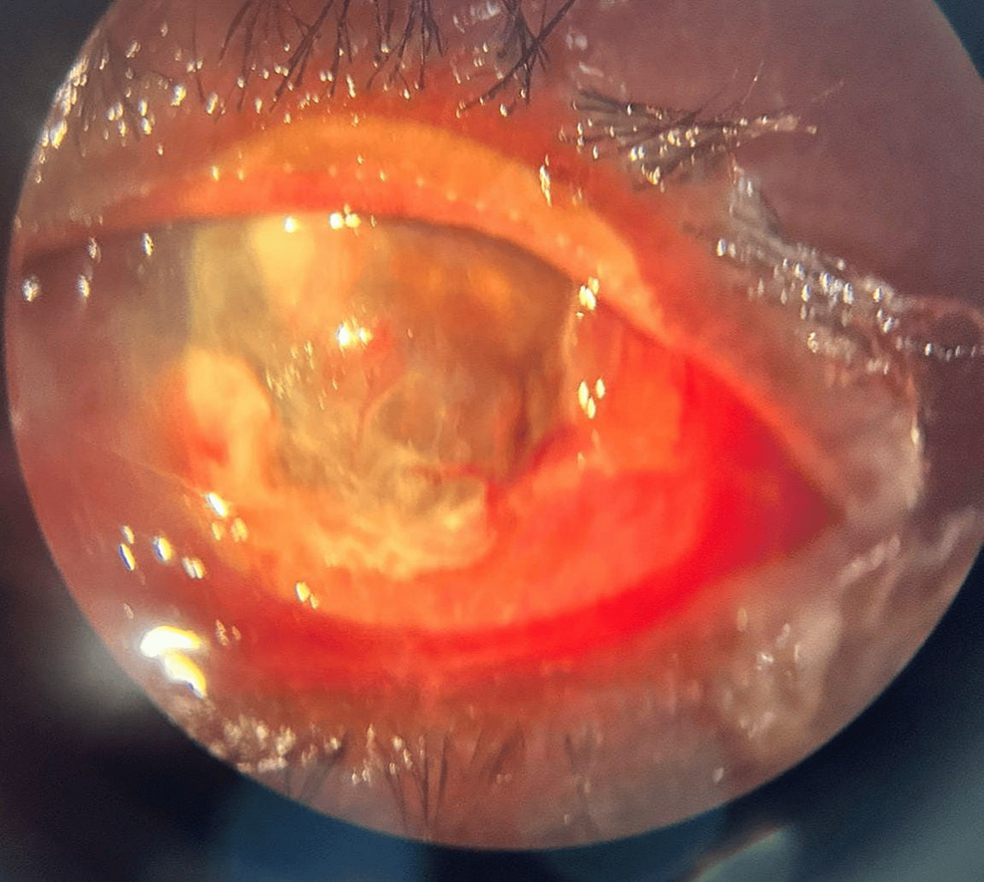
Image of the right eye showing matting of eyelids, severe conjunctival chemosis, diffuse corneal haze with exudates, and diffuse blood staining of the cornea

A 75-year-old male, diagnosed with dengue fever one week prior, presented with a sudden onset of severe pain and acute vision loss in his right eye. Initial blood tests upon hospital admission showed a platelet level of 35,000/mm³ and a positive NS1 IgM antigen test, confirming dengue fever. Supportive treatment was initiated, which involved intravenous fluids, broad-spectrum intravenous antibiotics, and platelet transfusion. On the fourth day of admission, the patient developed a sudden onset of severe pain and acute vision loss with associated discharge in his right eye, which did not respond to medications. The visual acuity was recorded as no perception of light in the right eye, while the left eye maintained 6/6 vision. Slit-lamp examination revealed significant matting of the eyelids accompanied by pronounced conjunctival chemosis. There was diffuse corneal haze and blood staining and exudates, obscuring the visualization of the remainder of the anterior segment (Figure 7).

**Figure 8:**
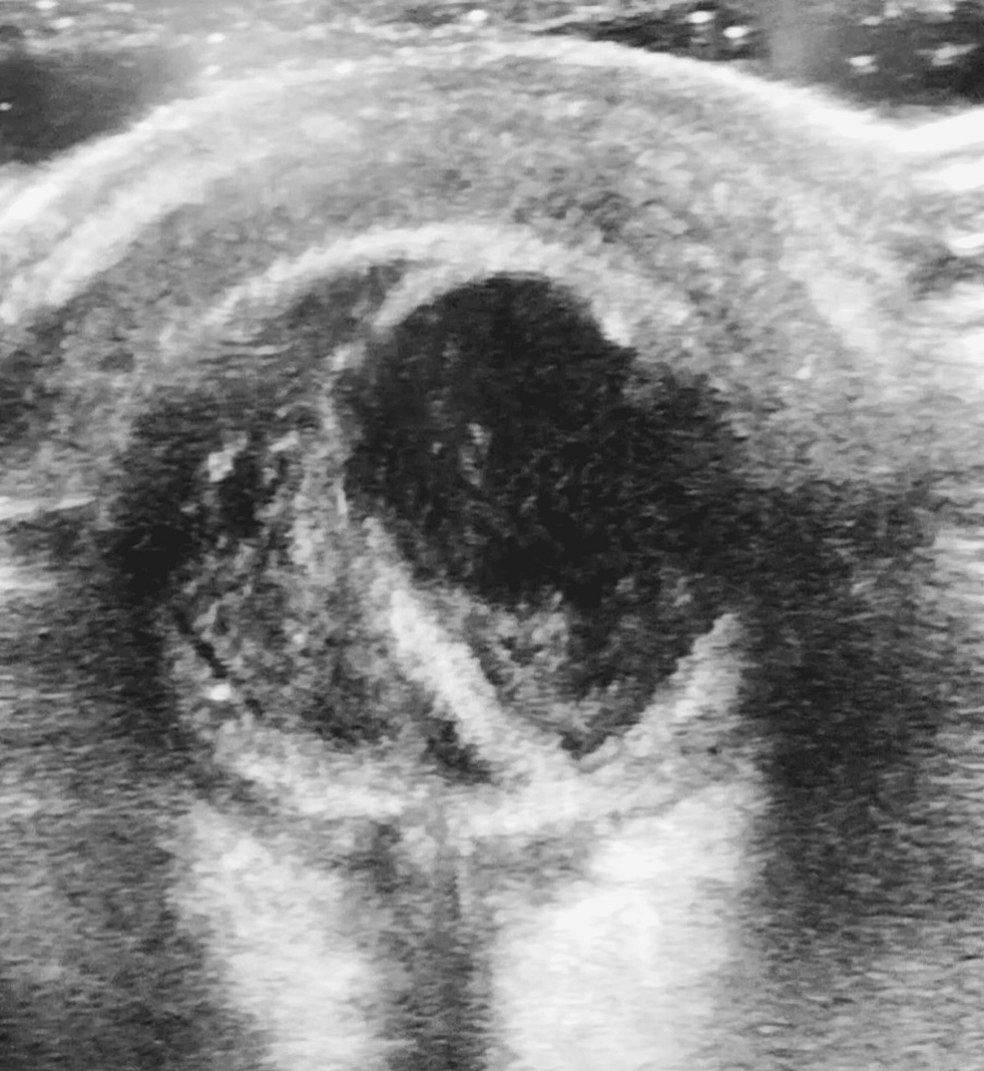
Ultrasound B-scan findings The image showed scleral thickening with a detached retina and vitreous echoes suggestive of panophthalmitis

A culture swab from the affected eye tested positive for Bacillus cereus. B-scan ultrasonography of the right eye demonstrated scleral thickening, a detached retina, and multiple vitreous echoes indicative of panophthalmitis (Figure 8). Despite receiving aggressive treatment with intravenous antibiotics, the patient had to eventually undergo evisceration with a conformer in situ and was discharged in stable condition. 

## Discussion

Dengue fever is typically characterized by fever, muscle pain, and rash, with severe cases posing life-threatening complications such as hemorrhages, shock, and, rarely encephalitis or myocarditis [1]. Ocular involvement in dengue, though rare, can manifest as uveitis, intra-retinal hemorrhages, vasculitis, optic neuropathy, choroidal effusions, and most severely, panophthalmitis [1]. The exact etiology of these ocular complications remains unclear, with proposed mechanisms including immune-mediated processes and factors like low albumin levels and reduced white blood cell count [3]. Dengue antibodies may cross-react with endothelial cells, compromising the blood-ocular barrier and allowing microorganisms to enter the eye, potentially leading to septicemia and focal infection near retinal vessels [3].

A few case reports have been published in the literature. For instance, a study by Saranappa et al. described a six-year-old patient who developed unilateral dengue panophthalmitis during the critical phase of the illness, resulting in permanent blindness [8]. Kamal et al. discussed the case of a patient with dengue hemorrhagic fever who subsequently developed panophthalmitis in the right eye, and the causative bacterium Bacillus cereus was isolated from the eviscerated sample [9]. Sriram et al. presented a case of a 25-year-old male who developed bilateral panophthalmitis as an initial manifestation of dengue fever. Ophthalmic complications, including uveitis and retinal issues, highlight the importance of early recognition and treatment to preserve vision and reduce morbidity [10].

Panophthalmitis represents a rapidly progressing suppurative process involving the retina, choroid, and sclera, leading to proptosis and restricted extraocular muscle movement [11]. Advanced stages may feature chemosis, increased proptosis, corneal infiltrates, and retinal detachment, culminating in blindness [3]. Proptosis in dengue fever may occur due to retrobulbar hemorrhage or panophthalmitis [3,12]. While ocular involvement in dengue is rare, it warrants consideration due to its potential for permanent disability. Dave et al. concluded that the definitive detection of dengue virus RNA in their series of cases, considering the established pathophysiology of systemic dengue, implies a direct influence of dengue virus on intraocular tissues, potentially contributing to the rapid advancement of ocular infections to panophthalmitis [4].

Arya et al. presented a case report of panophthalmitis associated with scleral necrosis in dengue hemorrhagic fever, which proposed that steroids play a crucial role in managing patients with dengue-related ocular manifestations and impaired vision. However, when proptosis arises from hemorrhage or endophthalmitis in individuals with dengue fever, achieving a favorable prognosis for vision and globe salvage is notably challenging [6]. Tripathy et al. reported that evisceration is frequently selected as the preferred surgical intervention for blind, painful eyes affected by endophthalmitis and panophthalmitis. The residual scleral shell is theorized to act as a barrier against contiguous posterior infection spread. This procedure typically involves the insertion of an orbital implant within the scleral shell to enhance cosmetic rehabilitation of the socket in the postoperative period [13].

## Conclusions

This case series underscores the rare occurrence of culture-positive panophthalmitis in the context of dengue fever, emphasizing the importance of early diagnosis and intervention to mitigate severe ocular involvement. Healthcare providers should remain vigilant of ocular complications in dengue patients and ensure prompt treatment initiation. Comprehensive ophthalmic evaluation and timely intervention are crucial in managing individuals presenting with dengue-associated ocular symptoms to prevent irreversible visual impairment. Ophthalmologists play a pivotal role in recognizing and managing these potentially sight-threatening complications.
